# Scoliosis in patients with Prader Willi Syndrome – comparisons of conservative and surgical treatment

**DOI:** 10.1186/1748-7161-4-10

**Published:** 2009-05-06

**Authors:** Hans-Rudolf Weiss, Deborah Goodall

**Affiliations:** 1 Koob-Scolitech, Huehnerhof 100, D-55568 Abtweiler, Germany; 2 ERS, Ealing Hospital, Uxbridge Road, Southall, UB1 3HW, London, UK

## Abstract

In children with Prader Willi syndrome (PWS), besides growth hormone (GH) therapy, control of the food environment and regular exercise, surgical treatment of scoliosis deformities seems the treatment of choice, even though the risks of spinal surgery in this specific population is very high. Therefore the question arises as to whether the risks of spinal surgery outweigh the benefits in a condition, which bears significant risks per se. The purpose of this systematic review of the Pub Med literature was to find mid or long-term results of spinal fusion surgery in patients with PWS, and to present the conservative treatment in a case study of nine patients with this condition.

Types of studies included; all kinds of studies; retrospective and prospective ones, which reported upon the outcome of scoliosis surgery in patients with PWS.

Types of participants included: patients with scoliosis and PWS.

Type of intervention: surgery.

Search strategy for identification of the studies; Pub Med; limited to English language and bibliographies of all reviewed articles.

Nine patients with PWS from our data-base treated conservatively have been found, being 19 years or over at the time this study has been performed. The results of conservative management are described and related to the natural history and treatment results found in the Pub Med review.

From 2210 titles displayed in the Pub Med database with the key word being "Prader Willi syndrome", 5 different papers were displayed at the date of the search containing some information on the outcome of surgery and none appeared to contain a mid or long-term follow-up. The PWS patients treated conservatively from our series all stayed below 70° and some of which improved.

If the curve of scoliosis patients with PWS can be kept within certain limits (usually below 70 degrees) conservatively, this treatment seems to have fewer complications than surgical treatments. The results of our retrospective study of nine patients demonstrate that scoliosis in this entity plays only a minor role and surgery is unnecessary when high quality conservative management exists.

There is lack of the long follow-up studies in post-surgical cases in patients with PWS and scoliosis. The rate of complications of spinal fusion in patients with PWS and scoliosis is very high and the death rates have been found to be higher than in patients with Adolescent Idiopathic Scoliosis (AIS). The long-term side-effects of the intervention are detrimental, so that the risk-benefit ratio favours the conservative approaches over spinal fusion surgery.

## Background

**Figure 1:**
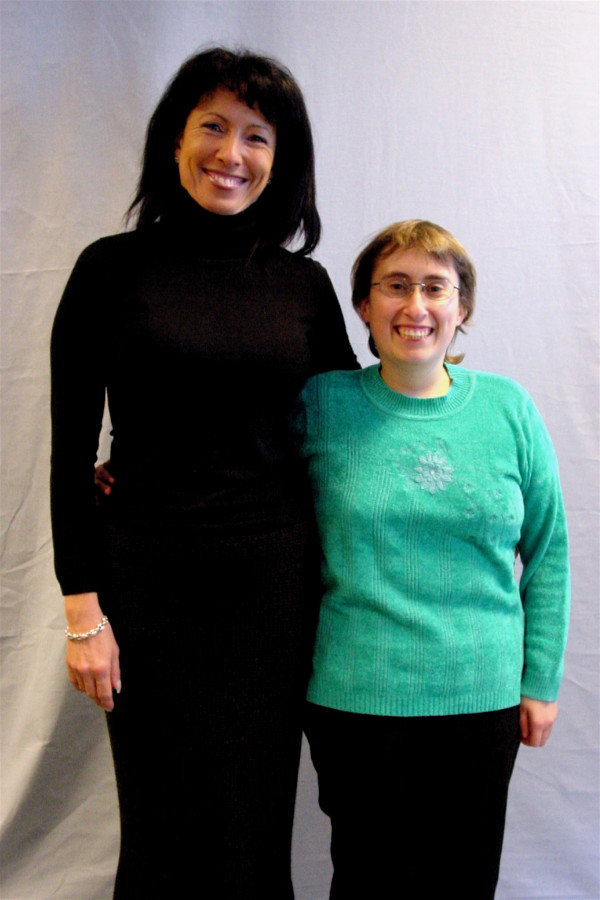
**Short statured Patient with only little overweight according to regular exercising**. This 24 year old woman with PWS is short statured, compared to her mother next to her on this picture. She has been very obese, while under brace treatment, however with good intake control of food, regular exercising and after having gone through 3 phases of in-patient rehabilitation during the time of brace treatment, she does not seem to be overweight now.

As pointed out by Molinas et al. [1] the rare genetic neurodevelopmental disorder called Prader-Willi syndrome (PWS), related to the lack of expression of paternal genes in the q11–q13 region of chromosome 15 was first described in 1956 [2]. The incidence of PWS is estimated to be about one out of 25000 births [3-5], most cases are sporadic and familial cases are rare. PWS infants exhibit severe hypotonia already present at birth that partially improves, explaining in part suckling and swallowing troubles, failure to thrive (i.e. failure to get weight besides normal or increased caloric intake) as well as delayed psycho-motor development [6-8]. After this initial phase of about 2 years, the most striking sign appears: early and severe obesity related to an increasing appetite and the decrease of satiety with an overwhelming eating obsession [9]. Furthermore, a wide range of associated troubles is reported in children, adolescents and adults: low and delayed motor and oral-motor skills, learning difficulties, growth hormone and sex hormones deficiencies probably due to an unknown hypothalamic dysfunction and resulting in short stature (Figure 1), increased body fat with a decreased lean mass, cryptorchidism and absence or blunted puberty [9]. Other common concerns include strabismus, scoliosis, osteoporosis, type 2 diabetes mellitus, hypertension, hypoventilation, heart failure, skin problems, sleep disturbances and skin picking (sometimes severe). Early diagnosis confirmed by genetic analysis combined with multidisciplinary care including hormonal therapies can dramatically smooth over PWS troubles and improve the quality of life of these patients and their family [10-14]. However, the constant and long-term need for food restriction, behaviour management and medical care may be stressful for PWS patients and family members [15,16].

Cassidy and Driscoll describe PWS to be a highly variable genetic disorder affecting multiple body systems whose most consistent major manifestations include hypotonia with poor suck and poor weight gain in infancy; mild learning difficulties, hypogonadism, growth hormone insufficiency causing short stature for the family, early childhood-onset hyperphagia and obesity, characteristic appearance, and behavioral and sometimes psychiatric disturbance. PWS is an example of a genetic condition involving genomic imprinting. It can occur by three main mechanisms, which lead to absence of expression of paternally inherited genes in the 15q11.2–q13 region: paternal microdeletion, maternal uniparental disomy, and imprinting defect [17].

Patel et al demonstrated that PWS patients may have significantly raised levels of the inflammatory marker hs-CRP and evidence of microcirculatory dysfunction, both of which are associated with coronary artery disease and early sudden death. The sinus node dysfunction may in itself be a risk factor for sudden cardiac death [18].

Some authors reported on primary lymphedema in a patient with PWS, an association, which is often missed [19].

Patients with PWS drawn from an adult and adolescent PWS clinic have a high rate of sleep-disordered breathing [20-22]. There is evidence that patients with PWS may have more nocturnal hypoventilation than a well-matched control group. [20]. Psychotic illness is strongly associated with PWS [23-25] and there are also intracranial abnormalities in individuals with PWS including ventriculomegaly (100% of individuals), decreased volume of brain tissue in the parietal-occipital lobe (50%), sylvian fissure polymicrogyria (60%), and incomplete insular closure (65%) [26]. Shu et al. [27] have measured the intelligence of subjects with PWS. Their intelligence tests showed full intelligence quotient = 52.0 +/- 7.6; verbal intelligence quotient equalled 55.9 +/- 8.77; performance intelligence quotient = 53.2 +/- 9.0. Chronic health status revealed that diabetes was prevalent among the older population. Their IQ was in the range of those with moderate learning difficulties.

Obesity and hypogonadism in patients with PWS are also frequently associated with slipped capital femoral epiphysis (SCFE) [28]. Slipped capital femoral epiphysis (SCFE) is a rare complication of growth hormone (GH) therapy as well [29].

The Italian National Survey for Prader-Willi syndrome offers an insight into the subject "causes of death in PWS": A total of 212 subjects had received GH treatment, of which 141 were still receiving therapy, while the remaining 71 had stopped. In children and adolescents (233 cases), 89 subjects had never undergone GH therapy. Eighteen PWS patients had died in the past 20 years. Obesity-related cardiovascular and respiratory diseases were the cause of death, both during childhood and after 18 years of age. Three children died suddenly whilst undergoing GH therapy. Respiratory infection and cardiac illness were the causes of death in two cases. There was no definitive cause of death found in the third case [30]. Several deaths have also been reported in children with Prader-Willi syndrome (PWS) following treatment with growth hormone (GH) [31]. The authors collected all of the reports of deaths in PWS children, both in treatment and non-treatment groups, analyzed the causes of the death and compared the two groups. Their analysis shows the high frequency of respiratory infections in both GH-treated and -untreated PWS children. The first nine months of GH treatment seems to be a high-risk period emphasising the need for comprehensive care before and during GH treatment [31]. According to Stevenson et al. [32] four reports of unexpected mortality due to gastric rupture and necrosis were found in 152 reported deaths, accounting for 3% of the causes of mortality. Four additional individuals were suspected to have gastric rupture.

Scoliosis seems a major concern for patients with Prader-Willi syndrome, and a regular (annual) systematic back examination is mandated [33]. The role of growth-hormone treatment on the natural history of scoliosis could not be determined, and careful monitoring during treatment is recommended [33]. Two types of scoliosis were identified by de Lind van Wijngaarden [34]: 1) "long C-curve type" scoliosis (LCS) and 2) "idiopathic" scoliosis (IS). Children were divided into age categories (infants: 0–3 years, juveniles: 3–10 years, adolescents 10–16 years). The prevalence of scoliosis was 37.5% and increased with age (infants and juveniles: 30%, adolescents: 80%), 44% of children with scoliosis had a Cobb angle above 20 degrees. Children with scoliosis were significantly older than those without. Children with LCS were younger and more hypotonic than those with IS. The prevalence of scoliosis, though, in PWS children is high (37.5%) and many children with scoliosis (13%) had undergone brace treatment or surgery [34].

Besides GH therapy from early childhood, control of the food environment (Figure 1) and regular exercise [35,36], surgical treatment of scoliosis deformities seems the treatment of choice [37], although the risks of spinal surgery in this specific population seems very high [38-40]. Therefore the question arises as to whether the risks of spinal surgery outweigh the benefits in a condition which already presents with significant risks.

In order to promote an intervention for a specific condition, it must be demonstrated that; 1) the natural history of the condition is undesirable; 2) the intervention alters this natural history in a favourable and reproducible manner; 3) the complications are minimal and 4) the long term side-effects of the intervention are not detrimental, so that the risk-benefit ratio favours the intervention over the condition's natural history [41].

As already discussed, the natural history of PWS is considerably undesirable. However scientifically we have to demonstrate that the intervention (surgery) alters the natural history of PWS in a favourable and reproducible manner. It is necessary to demonstrate that the long term side-effects of spinal fusion are not detrimental, so that the risk-benefit ratio favours the intervention over the condition's natural history, but the rate of complications has been revealed to be relatively high.

Purpose of this systematic review of the Pub Med literature was to find mid or long-term results of spinal fusion surgery in patients with PWS.

## Methods

### Exclusion and inclusion criteria for the selection of studies in this review

#### Types of studies included

all kinds of studies, retrospective and prospective ones, obviously reporting on the outcome of scoliosis surgery in patients with PWS have been taken into account.

#### Types of participants included

patients with scoliosis and PWS.

### Type of intervention: surgery

*Search strategy for identification of the studies*; Pub Med; limited to English language and bibliographies of all reviewed articles, where appropriate.

The search strategy included the terms; 'Scoliosis'; 'Prader Willi syndrome'; 'spine surgery'; 'scoliosis surgery'; 'spondylodesis'; 'spinal instrumentation' and 'spine fusion'.

#### Study selection

An electronic search was performed and the studies were selected based on title, abstract and key words. When appropriate a full copy of the articles was printed to determine whether or not they met the inclusion criteria. Additionally, the references of all included articles were checked for further papers that might meet the inclusion criteria.

A case study of patients with this condition has been investigated to estimate as to whether Prader-Willi patients with scoliosis may benefit from conservative scoliosis management. Nine Patients with this condition have been found in our out-patient database. Five of these patients (3 girls, two boys) today are 19 years and older and therefore are without any significant residual growth. Average Cobb angle was 47 degrees (34 – 66 degrees), average observation time was 6.4 years.

## Results

On the 18^th^ of November, when the search of literature was performed 2210 titles displayed in the PUB Med database with the key word being "Prader Willi syndrome", only.

1279 titles displayed with the key words "Prader Willi syndrome" and "Symptoms" and the most important titles are cited in the background section.

5 different papers are displayed with respect to treatment outcomes [33,37,38,42-44] and none appears to contain at least a mid- or long-term follow-up.

Accadbled et al. [38] report on 16 patients (3 males, 13 females) with scoliosis and PWS, who were operated on between 1974 and 2004. Mean age at scoliosis diagnosis was 6.2 years (range 0.5–13.5). Mean age at surgery was 12.3 years (range 5–15). Mean follow-up was 5.4 years (range 2–18). There were nine major complications (4 severe kyphosis above fusion, 2 deep infections, 1 transient paraplegia, 1 pseudarthrosis, 1 delayed wound healing). The 4 kyphosis required re-operation, 3 of which were complicated with permanent spinal cord injury. Minor complications affected six patients. The authors concluded: Scoliosis surgery is frequently necessary in PWS and is associated with high rate of complications. These are often related to specific features of this syndrome the surgeon should recognize and consider [38].

In another study only one of 92 patients reported to have had spinal fusion had PWS [44]

One study, which was not displayed in our search for outcomes of spinal fusion in patients with PWS is a case report only of one patient with Prader Willi syndrome having had spinal surgery [37].

**Figure 2:**
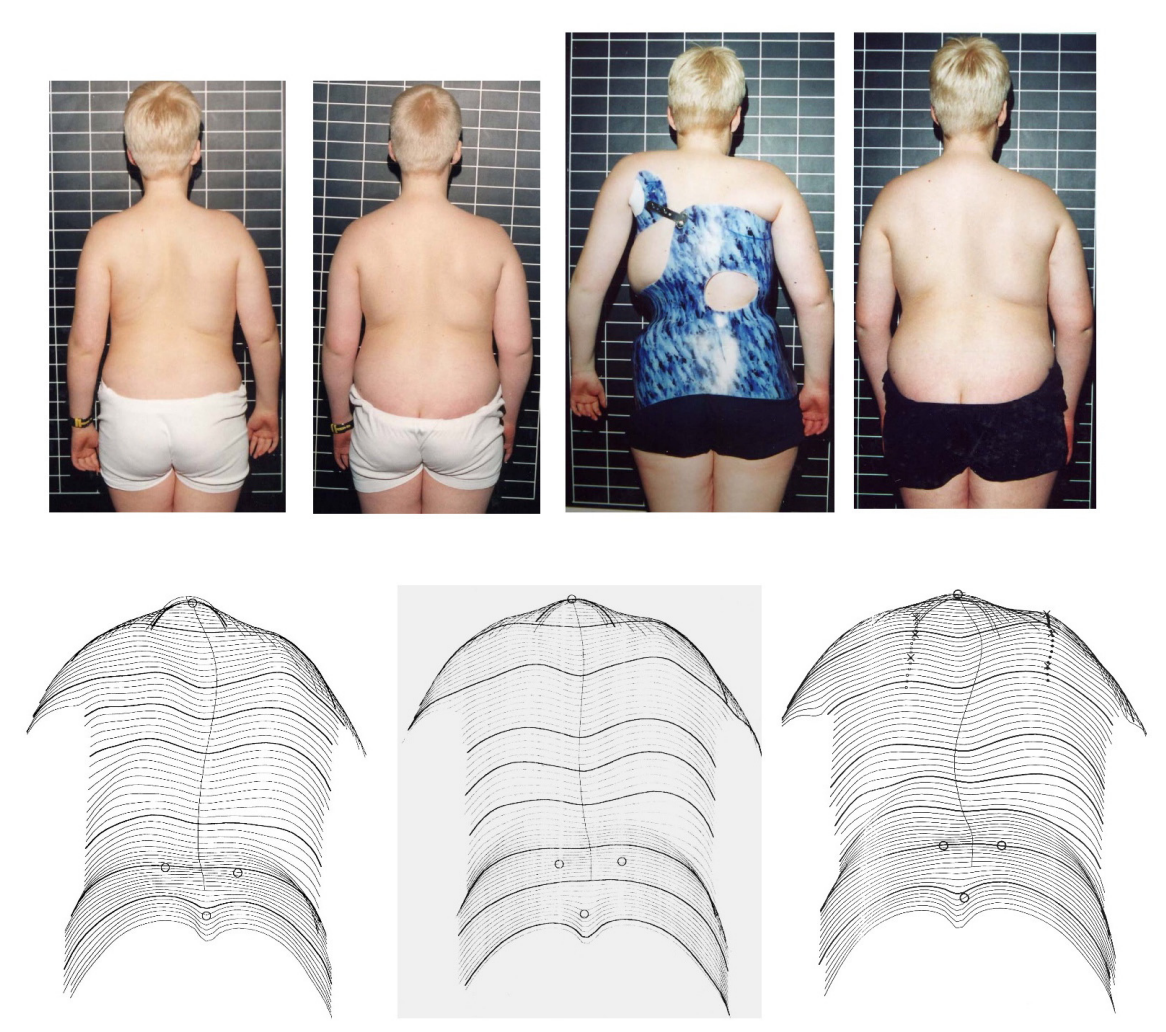
**PWS patient non-compliant to brace treatment**. This boy was not able to come across with his overwhelming eating obsession. He also was not compliant and therefore had a progression to 70° in the end. Left the patient at the start of treatment, intermediate result in the middle and final result at 19 years on the right. The last brace can be seen on the upper line.

Nine patients with PWS from our data base treated conservatively have been found, being 19 years or over at the time this study has been performed. The results of conservative management are described and related to the natural history and treatment results found in the Pub Med review. Two of the five patients being 19 years or over progressed. Average Cobb angle after follow-up was 52 degrees. No progression beyond 70 degrees has been found after cessation of growth. In one patient the curve deteriorated in response to this patient reducing the brace wearing times and therefore this negative result was clearly due to non-compliance (Figure 2).

## Discussion

Conservative management can prevent curve progression in patients with PWS and should to be regarded to be indicated for this patient group.

But specifically scoliosis in the case of a PWS seems to be less malignant than in other conditions. These patients do not tend to report to suffer from cosmetic concerns from the deformity and therefore rarely opt for surgery, which in patients with Adolescent Idiopathic Scoliosis (AIS) has to be regarded to be the main indication for spinal fusion [45-47].

Considering the fact that the complications of patients with PWS undergoing this surgery are high [38,41,45-47] and the health related benefits of such surgery in this population remain controversial [48-50], the indication for conservative treatment seems safer than spinal fusion surgery in this specific population. In patients with PWS clinical appearance is not the main reported problem by patients [51] with the exception of being overweight and this is why a cosmetic surgical intervention is not specifically indicated in this group in view of the significant complications [45,38].

**Figure 3:**
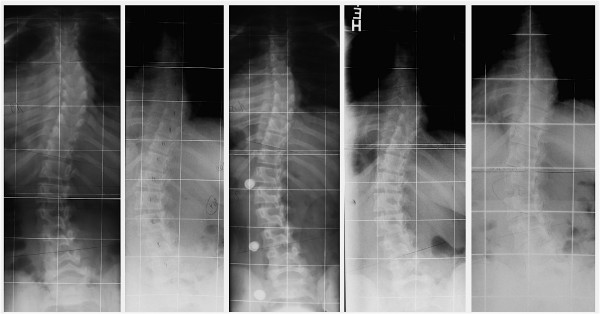
**PWS patient with improvement of scoliosis after cessation of brace treatment**. This patient has been treated with 5 braces after she has been progressive in early adolescence. She was compliant and made 3 courses of rehabilitation and at final follow-up, three years after brace weaning a stable improvement of the curve has been documented. Left: First presentation at the age of 11 years with 19° thoracic and 17° lumbar, 2^nd^. from left: Brace indication at 12 years with 30° thoracic and 40° lumbar, middle: In brace correction with 17° thoracic and 25° lumbar, 2^nd^. from right: At weaning at the age of 20 years with 29° thoracic and 37° lumbar and on the right: Four years without brace at 24 years with 24° thoracic and 30° lumbar. During the last 4 years the woman lost weight.

 In this present study only one patient showed a significant progression to 70 degrees due to non-compliance and psychiatric problems (Figure 2). The remaining patients of this study have been kept within the ranges recorded on initial assessment pre-bracing and exercises and one particular patient improved significantly at final follow-up at the age of 24 years (Figure 3).

If the curve of scoliosis patients with PWS can be kept within certain limits (usually below 70 degrees) conservative treatment should be the preferred treatment option, considering the lack of evidence there is for the long term outcome of surgery and the high risks of this intervention. When treated conservatively according to the latest standards [52], scoliosis in patients with PWS seems a relatively benign condition (Figure 3).

Of course we cannot compare our sample of patients to the sample of the series treated surgically by Accadbled [38], whose average Cobb angle was > 70° and we do no not intend to compare. Nevertheless, it seem inappropriate to operate on this patient population just for improving the Cobb angle even if the patients have Cobb angles of > 70° at average, when there is no evidence, that the long-term outcomes of surgery are superior to the untreated condition in the long-term.

Scoliosis rehabilitation [52-54] demonstrated to be helpful in activating the PWS patients and this seems essential in view of the fact, that exercising fosters weight control and physical fitness [35,36]. Studies of operated PWS patients followed up into late adulthood are necessary, before a medical indication for surgery can be established in the population having high risks when undergoing surgery.

## Conclusion

1. There is no long-term follow up on treatment outcomes available for PWS and scoliosis.

2. The rate of complications (including death) of spinal fusion in patients with PWS and scoliosis is very high.

3. The long-term side-effects of the intervention are detrimental, so that the risk-benefit ratio favours the conservative approaches over spinal fusion surgery.

## Consent

Written informed consent was obtained from the patients for publication of their cases and any accompanying images.

## Competing interests

The authors declare that they have no competing interests.

## Authors' contributions

HRW: manuscript writing, Pub Med research, Patient acquisition, Patient data base, database searches and Figures. DG: copyediting.
